# The Interplay of GPER1 with 17β-Aminoestrogens in the Regulation of the Proliferation of Cervical and Breast Cancer Cells: A Pharmacological Approach

**DOI:** 10.3390/ijerph191912361

**Published:** 2022-09-28

**Authors:** Mariana Segovia-Mendoza, Elahe Mirzaei, Heriberto Prado-Garcia, Luis D. Miranda, Alejandra Figueroa, Cristina Lemini

**Affiliations:** 1 Departamento de Farmacología, Facultad de Medicina, Universidad Nacional Autónoma de México, Av. Universidad No. 3000, Ciudad Universitaria, Ciudad de México 04510, Mexico; 2 Instituto Nacional de Medicina Genómica, Col. Arenal Tepepan, Ciudad de México 14610, Mexico; 3 Laboratorio de Onco-Inmunobiologia, Departamento de Enfermedades Crónico-Degenerativas, Instituto Nacional de Enfermedades Respiratorias Ismael Cosio Villegas, Calzada de Tlalpan 4502, Col. Sección XVI, Ciudad de México 14080, Mexico; 4 Instituto de Química, Universidad Nacional Autónoma de México, Circuito Exterior S.N., Ciudad Universitaria, Ciudad de México 04510, Mexico

**Keywords:** G-protein-coupled estrogen receptor (GPER1), 17β-aminoestrogens (17β-AEs), molecular docking, cell viability, cancer cells

## Abstract

The G-protein-coupled receptor for estrogen (GPER1) is a transmembrane receptor involved in the progression and development of various neoplasms whose ligand is estradiol (E2). 17β-aminoestrogens (17β-AEs) compounds, analogs to E2, are possible candidates for use in hormone replacement therapy (HRT), but our knowledge of their pharmacological profile is limited. Thus, we explored the molecular recognition of GPER1 with different synthetic 17β-AEs: prolame, butolame, and pentolame. We compared the structure and ligand recognition sites previously reported for a specific agonist (G1), antagonists (G15 and G36), and the natural ligand (E2). Then, the biological effects of 17β-AEs were analyzed through cell viability and cell-cycle assays in two types of female cancer. In addition, the effect of 17β-AEs on the phosphorylation of the oncoprotein c-fos was evaluated, because this molecule is modulated by GPER1. Molecular docking analysis showed that 17β-AEs interacted with GPER1, suggesting that prolame joins GPER1 in a hydrophobic cavity, similarly to G1, G15, and E2. Prolame induced cell proliferation in breast (MCF-7) and cervical cancer (SIHA) cells; meanwhile, butolame and pentolame did not affect cell proliferation. Neither 17β-AEs nor E2 changed the activation of c-fos in MCF-7 cells. Meanwhile, in SIHA cells, E2 and 17β-AEs reduced c-fos phosphorylation. Thus, our data suggest that butolame and pentolame, but not prolame, could be used for HRT without presenting a potential risk of inducing breast- or cervical-cancer-cell proliferation. The novelty of this work lies in its study of compound analogs to E2 that may represent important therapeutic strategies for women in menopause, with non-significant effects on the cell viability of cancer cells. The research focused on the interactions of GPER1, a molecule recently associated with promoting and maintaining various neoplasms.

## 1. Introduction

The G-protein-coupled receptors (GPCRs) belong to a family of seven transmembrane (7TM) receptors, which are expressed in a variety of tissues that govern a large number of non-genomic pathways [1]. 17β-estradiol (E2) mediates critical physiological processes through its binding with nuclear estrogen receptors (ERα and ERβ) [2]. Because of this, estrogen signaling has been recognized in multiple pathological and biological processes, including cancer [3]. Regardless of the genomic regulation of classic ERs, E2 binds to the G-protein-coupled estrogen receptor (GPER30/GPER1) and mediates different non-genomic events, including calcium mobilization, adenylate cyclase, tyrosine-protein kinase (Src), the cleavage of matrix metalloproteinases, the transactivation of the epidermal growth factor receptor (EGFR), and the activation of ERK1/2, Hippo/YAP/Taz pathways, among others [4,5,6]. GPER1 is involved in many processes, such as reproduction, cell proliferation, metabolism, mineral bone processes, and the cardiovascular, nervous, and immune systems [7,8,9]. GPER1 was first identified as an orphan receptor; later, it was demonstrated to mediate E2 signaling [10,11,12]. In the context of cancer, GPER1 may play important roles in regulating cellular proliferation, increases in tumor size, distant metastasis, and tumor recurrence [13,14]. Recently, different studies have exhibited its significant role in various types of cancer [15,16,17]. GPER1 has even been proposed to be a poor prognostic marker in different kinds of cancer [18,19]. Therefore, there has been substantial research into the identification of synthetic compounds that function as agonists or antagonists of GPER1. Different molecules with antagonist activity of nuclear receptors can serve as GPER1’s agonists, suggesting their particular role in different pathological scenarios. Based on these findings, GPCRs, including GPER1, are considered to be promising therapeutic targets for treating diverse tumors [16,20].

In the search for valuable candidates for hormone replacement therapy (HRT) in menopause, our working group has, for some years, studied different synthetic compounds with estrogenic activity known as 17β-aminoestrogens (17β-AEs). These compounds have moderate anticoagulant effects in rats and mice [21]. These effects are opposite to the pro-coagulant effects exhibited by E2 and other synthetic estrogens in clinical use that are potentially thrombogenic [22]. High doses of 17β-AEs administered in ovariectomized rats are not pro-thrombotic, indicating that they could be a helpful alternative in clinical situations with a predisposition to thrombosis. Various 17β-AEs, such as prolame, butolame, and pentolame, have shown lower estrogenic activity than the natural hormone E2 in different in vivo and in vitro models, indicating a low potential for toxicity in both the uterus and the breasts [23,24]. Recent works have also found that 17β-AEs produce neuroprotective effects in ovariectomized rats through the modulation of antidepressant, anxiolytic, and mnemonic effects [25,26], which are equivalent to or greater than the effects induced by E2. The above characteristics make 17β-AEs valuable candidates for HRT. However, until now, there has been no research into the effects of 17β-EAs in breast and cervical cancer cells due to their binding to GPER1.

Cervical cancer is one of the most common gynecological women’s cancers. Recent studies have suggested that cervical cancer’s persistence and malignant progression can be modulated by estrogenic signals [27,28,29,30,31]. In cervical cancer, the presence of GPER1 has also been reported recently, and is related to carcinogenesis markers and the repression of tumor suppressor proteins such as p16 and p53 [18]. However, there are few reports on the molecular effects of GPER1 in this type of cancer. Remarkably, GPER1 transactivates with the epidermal growth factor receptor (EGFR), stimulating downstream signaling pathways [32,33]. In this sense, the presence of EGFR has also been linked with primary and metastatic cervical cancer [34]. Therefore, evaluating the activation of GPER1 in this type of cancer is also important. On the other hand, in breast cancer, the presence of GPER1 has been associated with both hormone-dependent and independent types of cancer, such as luminal types (which are dependent on ER signaling), epidermal growth factor receptor 2 (HER2), and triple-negative types [35,36,37]. Different articles have shown that GPER1 can confer resistance to conventional estrogen-dependent breast cancer treatment [38].

Several estrogen and anti-estrogen compounds display different binding affinities for GPER1 and nuclear ERs [39,40]. Thus, structure-based drug design and virtual ligand screening have led to the identification of selective ligands to GPER1 [40]. Some years ago, the discovery and characterization of G-1 ([1-(4-[6-bromobenzo-(1,3)-dioxol-5-yl]-3a,4,5,9b-tetrahydro-3H-cyclopenta-[c]-quinolin-8-yl)-ethanone]) [41], G-15 ([4-(6-bromo-benzo-[1,3]-dioxol-5-yl)-3a,4,5,9b-tetrahydro-3H-cyclopenta-(c)-quinoline]) [42], and G36 ([4-(6-bromo-benzo-[1,3]-dioxol-5-yl)-8isopropyl-3a,4,5,9b-tetrahydro-3H-cyclopenta-(c)-quinoline]) [43], which act as selective agonists or antagonists to GPER1, respectively, contributed to the development of new strategies for the characterization of GPER1 signaling. In this sense, different molecular coupling analyses (docking) have primarily been considered. The characteristics typically considered in these studies are 2D structural patterns based on shape analogies, pharmacophore-based motifs, including hydrogen-bond donors/acceptors, and hydrophobic and π interactions, among others [40].

Thus, this study aimed to perform molecular simulation studies of 17β-AEs with GPER1 and corroborate their proliferative actions in biological assays in breast and cervical cancer cell lines to assess whether these compounds represent potential therapeutic options in HRT for women in menopause, avoiding cancer cell development and proliferation.

## 2. Materials and Methods

### 2.1. Reagents

Estradiol (E2, 1,3,5(10)-estratrien-3,17β diol) and sulforhodamine B (SRB) were purchased from Sigma Aldrich (St. Louis, MO, USA). The 17β-AEs, prolame [17β-(3-hydroxy-1-propylamine)-1,3,5(10)-estratrien-3-ol)], butolame [17β-(3-hydroxy-1-butylamine)-1,3,5(10)-estratrien-3-ol)], and pentolame [17β-(5-hydroxy-1-pentolamine)-1,3,5(10)-estratrien-3-ol)], were prepared from estrone according to previously reported methods [21,44]. G36 (4-(6-bromo-benzo[1,3]dioxol-5-yl)-8-isopropyl-3a,4,5,9b-tetrahydro-3H-cyclopenta quinoline) was synthesized and characterized according to the procedure reported by Denis et al. [43]. 

The compounds were synthesized following exactly the reported procedures [21,44]. E2, the 17β-aminoestrogens, and G36 were dissolved in absolute ethanol and used as a stock solution (Merck, Darmstadt, Germany). All cell culture reagents and media, as well as the molecular biology reagents, were purchased from Gibco (Invitrogen Corporation, Waltham, MA, USA).

Additionally, 7-Aminoactinomycin D (7-AAD) was acquired from BioLegend, San Diego, CA, USA, Phosflow Fix Buffer I and Phosflow Perm Buffer III were purchased from Becton Dickinson (BD) Biosciences, San Jose, CA, USA), and the rabbit anti-phospho c-fos (SER32) monoclonal antibody was acquired from Cell Signaling Technology, Danvers, MA, USA.

### 2.2. Molecular Modeling and Docking

To date, the crystallographic structure of GPER has not yet been fully determined. We prepared an in silico model to identify and describe the possible affinity and binding of the selected ligands with the GPER1 protein. The protein sequence was obtained from the UniProt database and submitted to the GPCR I-Tasser website [45], where the 3-D structure was constructed using the ten different template crystal structures most similar to the protein (4mbsA, 5t1aA, 5vblB, 6d26A1, 6wwzR, 3oduA, 6do1A, 5wb1A1, 4n6hA, and 2ziyA). ERRAT was used to assess the best model. (http://nihserver.mbi.ucla.edu/ERRAT/ (accessed on 6 July 2021)). The ChemBioDraw Ultra 12.0 program was used to draw the structures of the ligands. Geometric minimization was optimized at the AM1 semi-empirical level using Gaussian 03 software [46]. Subsequently, docking studies were carried out with the AutoDock 4.2 tool. Protein preparation, as polar hydrogens and Kollman charges, was calculated with Autodock Tools 1.5.1. Flexible residues were selected as Glue275, His 282, Phe278, Ile308, Vall 116, Met 133, Tyr123, Gln138, Phe 208, Ala209, Val 309, Asp 210, Phe 206, Phe314, and Leu 137. The grid box was fixed with x-66.711, y-66.605, z-54.007 a 0.375 Å grid spacing. The docking runs the 100 Lamarckian Genetic Algorithm. Finally, the energy values were calculated with a scoring function in Autodock.

### 2.3. Cell Culture

MCF-7 and SIHA cancer cell lines were acquired from ATCC (Manassas, VA, USA). The cells were seeded in RPMI media following instructions from the supplier. The media were supplemented with 5% heat-inactivated FBS (Hyclone Laboratories Inc. (Logan, UT, USA)) and 100 units/mL penicillin plus 100 µg/mL streptomycin, and maintained at 37 °C with a 5% atmosphere of CO_2_ and 95% humidity.

### 2.4. Cell Viability

The cells were seeded in 96-well culture plates at a density of 2500 cells per well; then, MCF-7 and SIHA cells were incubated in the absence (0.1% *v*/*v* ethanol) or presence of increasing concentrations of (1 × 10^−12^–1 × 10^−9^ M) of E2, prolame, butolame, and pentolame, either alone or combination with G36 (1 × 10^−6^ M), for 72 h.

Subsequently, the cell viability was quantified using the Sulforhodamine B assay; this assay was also used to evaluate cell proliferation [47]. For this purpose, the cells were fixed with 10% trichloroacetic acid for one hour at 4 °C, washed with water, and stained with sulforhodamine in acetic acid under agitation for 20 min. Finally, the cells were washed with 1% acetic acid twice; once the wells were dry, 10 mM Tris Base was added and they were read at 492 nM. Experiments were performed in triplicate on at least three different occasions.

### 2.5. Cell-Cycle Analysis

MCF-7 and SIHA cells were incubated with 1 × 10^−9^ M of E2, prolame, butolame, and pentolame, either alone or in combination with G36, for 72 h. After treatment, the cells were collected and washed with phosphate buffer (PBS) pH 7.2, fixed in 70% *v*/*v* ethanol, and stored at −20 °C. The samples were washed and incubated for cell-cycle analysis in a 0.1% *v*/*v* triton X-100 and 7-AAD (4 μL/1 × 10^6^ cells) solution in the dark at room temperature for 20 min. The DNA content was determined using a FACsCanto II flow cytometer (Becton Dickinson, San Jose, CA, USA). For cell-cycle analysis and the detection of the SubG0 peak, a total of 25,000 events from the 7AAD-area vs. the 7AAD-wide gate were acquired. The results were analyzed using Flow-Jo software (v.10 Becton Dickinson).

### 2.6. Phosphorylation Analysis

The phosphorylation of the oncogene c-fos (SER32) was determined in MCF-7 and SIHA cells by flow cytometry after six hours of incubation with Vh (Ethanol 0.1%) E2, 17-AEs alone, or in combination with G36. Briefly, the cells were trypsinized and washed with PBS. To evaluate the protein levels of the phosphorylated form of c-fos, the cells were treated with Phosflow Fix Buffer I/Phosflow Perm Buffer III (BD Phosphoflow) according to the manufacturer’s protocol. The cells were incubated with Phosflow Fix Buffer I at 37 °C for 10 min. Then, the cells were washed and incubated with Phosflow Perm Buffer III on ice for 30min. After permeabilization, the cells were washed with PBS/BSA (0.1%) and resuspended in 100 μL of rabbit anti-phospho c-fos (SER32) monoclonal antibody (dilution 1:1600, cat. no. C02-5348S, Cell Signaling Technology, Danvers, United States). After 45min of incubation with the primary antibody, the cells were washed and incubated with the Alexa 488 mouse anti-rabbit monoclonal antibody (Biologend, CA, USA) for 30 min. Cells were washed and resuspended in paraformaldehyde (1% *w*/*v*) to prepare for the flow cytometric analysis.

At least 10,000 events were acquired from the region of single tumor cells. The results were analyzed using Flow-Jo. The MFI values for phosphor c-fos were determined.

### 2.7. Statistical Analysis

The statistical differences among the groups were determined by employing a One-way ANOVA, followed by a Tukey test, *p* < 0.05. The specialized software package GraphPad Prism version 6 (San Diego, CA, USA) was used for the analysis. All values are expressed as the mean ± standard deviation (S.D.).

## 3. Results

### 3.1. Ligand Structures

**Figure 1:**
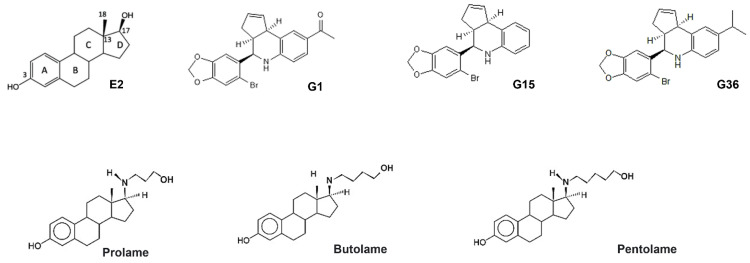
Chemical structure of E2, G1, G15, G36, prolame, butolame, and pentolame.

To determine the affinity of 17β-AEs to GPER1 and to perform the molecular simulation analyses, we used the structures of the previously reported agonists (E2, G1) and antagonists (G15, G36) of this receptor, as shown in Figure 1. 17β-AEs, prolame, butolame, and pentolame are compounds analogous to E2 and possess an amino group in the C17 position of the steroid nucleus, instead of the hydroxyl (OH) group, as is the case in E2 (Figure 1). They differ specifically in the length of the side chain substituent of the amino group at C17, which comprises three, four, or five methylene groups, respectively.

### 3.2. Molecular Docking Analysis

**Figure 2:**
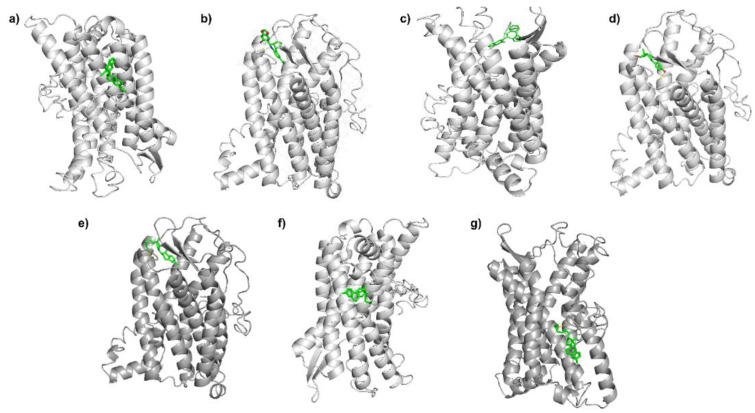
Gray ribbon cartoon of the binding conformations of GPER with (**a**) G36, (**b**) G1, (**c**) G15, (**d**) estradiol, (**e**) prolame, (**f**) butolame, and (**g**) pentolame.

The molecular docking results showed that the 17β-AEs interacted with the hydrophobic cavity of GPER1, where several already reported agonists (E2 and G1) and antagonists (G15 and G36) are coupled, as shown in Figure 2.

Table 1 shows the docking results, indicating the amino acid residues of each compound and highlighting the interaction that each compound established with the GPER1, according to the predictions of the in silico studies carried out in this work. In addition, the binding free energy (ΔG, kcal/mol) values for each tested compound were obtained from the molecular docking simulations, as shown in Table 2.

**Figure 3:**
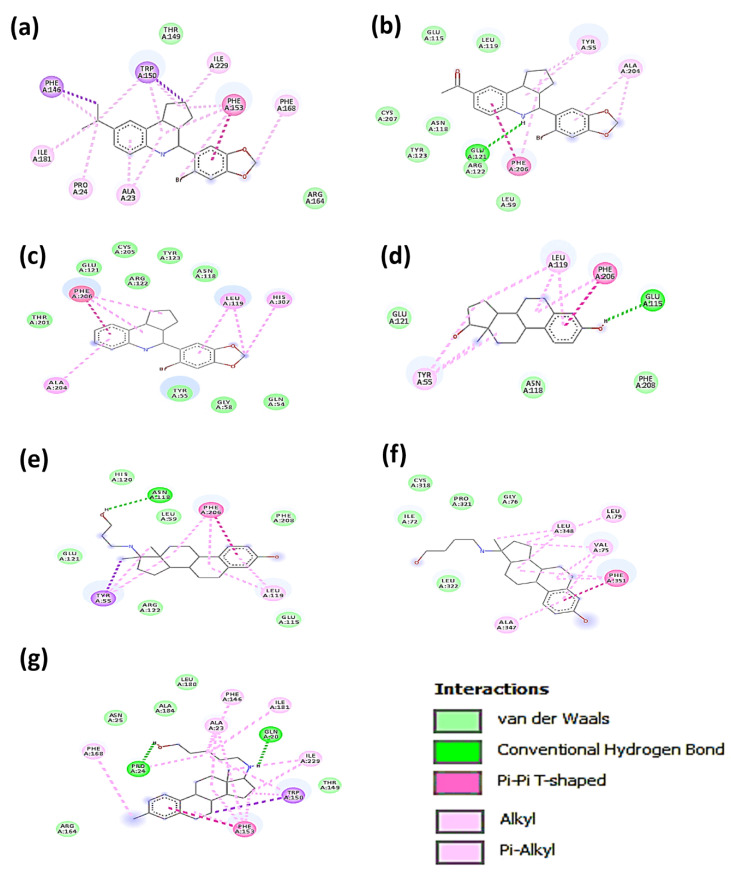
Representative amino acid interaction modes of (**a**) G36, (**b**) G1, (**c**) G15, (**d**) estradiol, (**e**) prolame, (**f**) butolame, and (**g**) pentolame with GPER1 derived from docking studies.

The binding amino acid motifs among GPER1 and the tested compounds are displayed in Figure 3. G36 presented many interactions, including π–π, π–alkyl, and alkyl. G1 and G15 were characterized mainly by Van der Waals forces interacting with GPER1. E2 had fewer interactions than G36, G1, and G15 (Table 1), but shared some binding amino acid motifs. G1 and G15 shared common Van der Waals interactions of asparagine (ASN) 118, arginine (ARG) 122, and tyrosine (TYR) 123 with GPER1. Both compounds interacted with leucine (LEU) 119, but, in the case of G1, it corresponded to a Van der Waals force, while in G15, LEU:119 exhibited a π interaction. Since G1 and G15 have many common interactions, they might hold the same binding cavity. Remarkably, E2 conserved the ASN:118 motif, as did G1 and G15. In addition, E2 also kept glutamic acid (GLU) 121 residue as a prevalent interplay with GPER1, like G15, and TYR:55, like G1.

Regarding 17β-AES, prolame presented three similar binding motifs with G15; GLU:121, ARG:122, and PHE:206. Notably, the PHE:206 motif was also shown in G1, G15, and E2. Prolame had an ASN:118 amino acid interaction—not in a hydrophobic sense, but rather as a hydrogen bridge. Prolame, G15, and E2 exhibited π interactions with leucine (LEU) 119.

The docking results show that butolame established interactions with GPER1 in different amino acid positions than those observed from G36, G1, G15, butolame and pentolame; see Table 1.

On the other hand, although the ΔG of pentolame was lower than that of G36, this compound had four amino acid motifs similar to G36: two of them were hydrophobic interactions (threonine (THR) 149 and ARG:164), one corresponded to a π interaction (PHE:153), and the other was an alkyl interaction (PHE:168), Figure 3 and Table 1.

For the subsequent analyses, E2 and G36 were used to compare the biological effects and pharmacological activity of 17β-AEs in two types of female cancer.

### 3.3. Cell Viability

The cell viability assays were performed at 24 and 72 h in breast and cervical cancer cells. In general, similar trends in cell viability were exhibited after 24 and 72 h of exposure to the compounds. Therefore, we focus on the effects obtained at 72 h to offer an overview of the longer term biological effects.

In MCF-7 cells, we found that E2 and prolame increased the fold cellular protein content in a similar range to E2 at 1 × 10^−9^ M and prolame at 1 × 10^−10^ M. It induced around 29% and 25% of the cell viability of MCF-7 cells, respectively, in comparison to the vehicle. Thus, these compounds induced cell proliferation Interestingly, butolame showed significant differences in diminishing cell viability, having a greater effect at the highest concentration (1 × 10^−9^ M). Butolame reduced cell viability by 32% and 28% compared to the E2 and prolame treatments, respectively. The treatment with pentolame did not show significant differences with respect to the vehicle or treatment with prolame or butolame. In addition, a marked decrease in cell viability was evident when cells were exposed to G36 (G). The decrease in cell viability caused by G36 was significant compared to the vehicle, E2, or the 17β-AEs administered independently.

In the combination of E2 plus G36 (E + G), we observed that G36 had a slight effect, antagonizing the proliferative effects of E2. In contrast, the difference observed under exposure to G36 alone was substantial and statistically significant, showing a 46% reduction with the presence of G36 alone compared to the different combinations of E2 plus G36.

**Figure 4:**
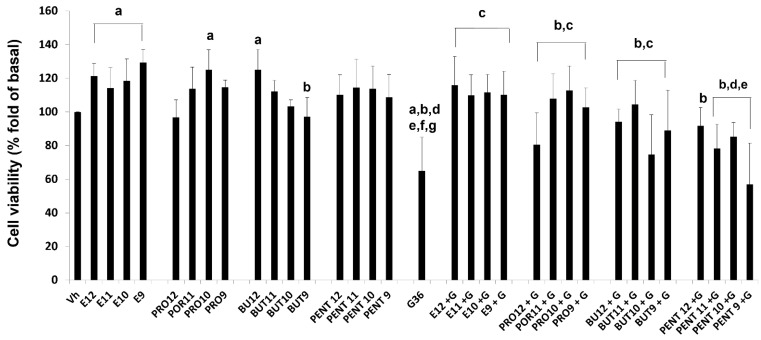
Effects on breast cancer cell viability of E2, prolame (PRO), butolame (BUT), pentolame (PENT), and G36, either alone or in combination with G36 (+G). A MCF-7 cell line was incubated for 72 h in the presence of ethanol (Vh 0.1%), estradiol (E2,), or 17β-AEs (1 × 10^−12^–1 × 10^−9^ M), either alone or in the presence of 1 × 10^−6^ M of G36 (G). Cell viability was evaluated using the sulforhodamine B method. Results are shown as the mean ± SD of sextuplicate determinations of three independent experiments. Data from vehicle-treated cells were normalized to 100. *p* < 0.05. Significant differences: a = vs. Vh; b vs. E2; c vs. G36; d vs. E + G; e vs. Pro; f vs. Pro + G; g vs. Pent + G. It should be noted that the treatments alone in a range of concentrations of 1 × 10^−12^–1 × 10^−9^ M, or in combination with G36 (1 × 10^−6^ M), are indicated on the X axis of the figure. We simplify the scientific notation for reasons of space; for example, E2 1 × 10^−12^ M = E12 and so on is used for each independent compound. In the case of G36, its concentration is not indicated on the axis of the figure; however, as highlighted above, the concentration of 1 × 10^−6^ M was used in all cases. The combination scheme of this compound is represented as follows: +G.

Comparing each combination of the 17β-AEs with each other, the results showed no differences among the combined treatments of E2 and G36 (E + G) and prolame plus G36 (PRO + G). We only found significant differences in the reduction of cell viability in the combinations of butoame plus G36 (BUT + G, 22%) and pentolame plus G36 (PENT + G, 53%), when compared with their corresponding combination in the estradiol plus G36 (E + G), (Figure 4). It should be noted that the antagonism of G36 is favored in the presence of pentolame, since this was the combination where the most significant reduction in cell viability was found.

**Figure 5:**
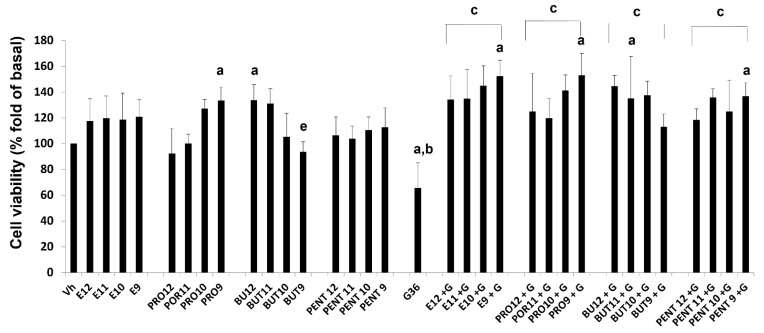
Effects on cervical cancer cell viability of E2, prolame (PRO), butolme (BUT), pentolame (PENT), and G36, either alone or in combination with G36 (+G). A SIHA cell line was incubated for 72 h in the presence of ethanol (Vh 0.01%), E2, or 17β-AEs (1 × 10^−12^–1 × 10^−9^ M), either alone or in the presence of 1 × 10^−6^ M of G36 (G). Cell viability was evaluated using the sulforhodamine B method. Results are shown as the mean ± SD of sextuplicate determinations of three independent experiments. Data from vehicle-treated cells were normalized to 100. *p* < 0.05. Significant differences: a vs. Vh: b vs. E2; c vs. G36; e vs. Pro; f vs. Pro + G; g vs. Pent + G. It should be noted that the treatments alone in the concentration range of 1 × 10^−12^–1 × 10^−9^ M, or in combination with G36 (1 × 10^−6^ M), are indicated on the X-axis. We simplify the scientific notation for reasons of space, for example using E2 1 × 10^−12^ M = E12 and so on for each independent compound. In the case of G36, its concentration is not indicated on the axis of the figure; however, as highlighted above, the concentration of 1 × 10^−6^ M was used in all cases. The combination scheme of this compound is represented as follows: +G.

In SIHA cells, we did not find differences in cell viability between the E2 treatment and the vehicle. In the case of 17β-Aes, prolame showed a concentration–response trend, increasing the cell proliferation by 27% and 33% only at the highest concentrations. Butolame had the opposite effect to prolame, significantly decreasing cell viability by 40% at the highest concentration. Finally, the treatment with pentolame did not show significant differences with respect to the vehicle, E2, prolame, and butolame. As was observed in MCF-7 cells, treatment with G36 significantly reduced the cell viability (35%) of SIHA cells (Figure 5).

Most of the combined schemes of E + G, PRO + G, BUT + G, and PENT + G induced cell proliferation only at high concentrations. However, any combined scheme showed significant differences concerning E2 or the 17β-Aes treatments independently, at each respective concentration. It should be noted that, for the G36 treatment, all the combinations showed statistically significant differences from the behavior exhibited by the G36 treatment alone (Figure 5).

### 3.4. Cell Cycle

To confirm the results obtained with the cell viability assays, and to clarify a possible mechanism of action of the treatments above, we evaluated the cell-cycle modulation by flow cytometry in breast and cervical cancer cells. Because we did not determine a similar or constant biological effect on cell viability after treatment with 17β-AEs with respect to E2 in the breast or cervical cancer cells, we decided to use the concentration of 1 × 10^−9^ M for E2 and the 17β-AEs in the rest of the experiments, since it is within the physiological ranges of E2 found in human serum [48].

**Figure 6:**
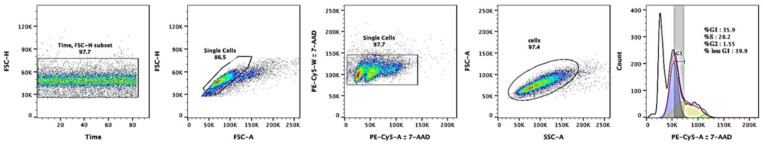
Representative gating strategy for cell-cycle analysis on SIHa cells treated with the G36 compound, which caused DNA fragmentation (SubG1 peak). Time vs. FSC h was used to monitor fluidic fluctuations. Then, singlets were selected using FSC-A vs. FSC h and 7-AAD-A vs. 7AAD-w. Single cells were analyzed and cell-cycle phases were calculated using the Jean–Dett–Fox model from Flow-Jo.

**Figure 7:**
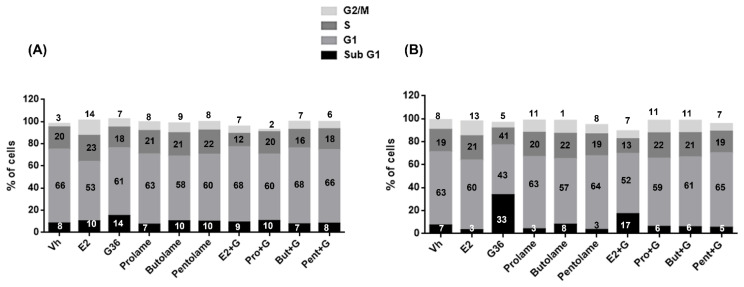
Effects of E2, prolame (PRO), butolme (BUT), pentolame (PENT), and G36, either alone or in combination with G36 (+G), on the cell cycle of the (**A**) MCF-7 and (**B**) SIHA cell line. Cells were incubated for 72 h in the absence (Vh) or presence of E2 or different 17β-AEs either alone or in combination with G36. Then, DNA content was analyzed as in Figure 6. Stacked columns show the mean percentages of cells in different cell-cycle phases for each treatment.

Figure 6 shows the gating strategy for the cell-cycle analysis. The cell-cycle profiles of the MCF-7 and SIHA cells treated for 72 h with the vehicle, E2, and G36, either alone or in combination, are displayed in Figure 7. Table 3 shows the percentages obtained from each cell-cycle phase for the different treatments in the MCF-7 line. G36 induced cell death, as evidenced by a significant increase in the SubG1 peak with respect to both the vehicle and the prolame treatment. Interestingly, when cells were exposed to prolame, the percentage of cells in the SubG1 phase was reduced, compared with the G36 treatment. In the G1 and S phases, no significant changes were observed with any of the treatments.

E2 induced a significant increase in the G2/M population compared to the vehicle. In contrast, the treatment with G36 reduced the percentage of cells in this phase, compared with the treatment with E2. These changes are in line with the results obtained from the cell viability experiments. Interestingly, adding G36 to E2 reduced the proportion of cells in the G2/M phase; see Figure 7A, Table 3.

Treatment with all the 17β-AES significantly increased the percentages of cells in the G2/M phase with respect to the vehicle or E2. In their combined schemes, the 17β-AES diminished the percentage of cells in this cell-cycle phase compared to the treatment with E2. Moreover, the combination of Pro + G was significantly different from the E + G treatment. All the combined schemes of 17β-AEs with G36 reduced the percentage of cells in the G2/M phase compared to E2. In addition, we observed that the 17β-AEs combined with G36 tended to reduce the percentages of cells in the G2/M phase, compared with their respective independent treatments; see Figure 7A, Table 3.

With respect to SIHA cells, the most remarkable increase in the SubG1 phase corresponds to treatment with G36. It should be noted that exposure to 17β-AEs, either alone or in combination, did not induce cell death. Exposure to E2, prolame, butolame, and pentolame alone or in combination with G36 showed a cell-cycle profile very similar to that of the vehicle-treated cells. In contrast, exposure to G36 significantly decreased the percentage of cells in the G1 phase, as shown in Figure 7B and Table 4.

### 3.5. Phosphorylation Analysis

We next evaluated whether the 17β-AEs modulate the active form of c-fos, which is regulated by GPER1, by analyzing the phosphorylation of c-fos at SER32 in breast and cervical cells by flow cytometry. It is worth mentioning that the phosphorylation status of c-fos was evaluated at one hour and at six hours after treatment. We will focus on the results obtained 6 h after exposure to the treatments, since we did not see significant changes at one hour (Supplementary Material Figure S1).

**Figure 8:**
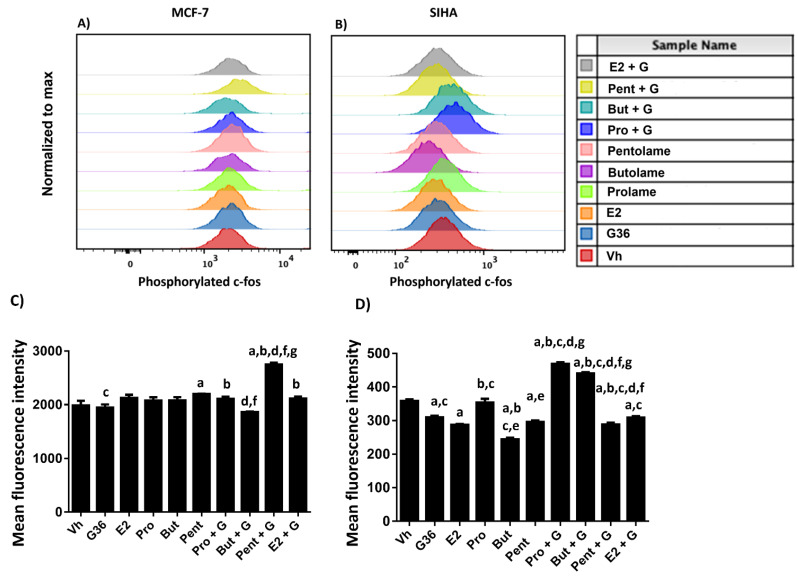
Analysis of the phosphorylation of c-fos in MCF-7 and SIHA cells. Histograms of a representative experiment of (**A**) MCF-7 and (**B**) SIHA cell lines. MFI values for each treatment are shown. Median fluorescence intensity (MFI) of c-fos in (**C**) MCF-7 and (**D**) SIHA cells are displayed. Bars represent data of at least three independent culture experiments expressed as the mean + S.D. *p* < 0.05. Statistical differences code: a vs. Vh; b vs. G36; c vs. E2; d vs. E + G; e vs. Pro; f vs. Pro+; g vs. Pent + G.

The results show that treatment with E2, prolame or butolame did not induce changes in c-fos phosphorylation in comparison to the vehicle; meanwhile, pentolame treatment induced significant phosphorylation in MCF-7 cells, but only in comparison to the vehicle, as evidenced by an increase in MFI values for phosphorylated c-fos. Regarding G36, this compound inhibited the c-fos phosphorylation by around 10% compared to E2 (Figure 8A,C).

The combinations of E + G, Pro + G, and Pent + G significantly increased c-fos phosphorylation with respect to the G36 treatment alone. Nevertheless, the Pent + G combination increased c-fos phosphorylation, not only in comparison with G36 but with respect to the other treatments. Interestingly, the But + G combination decreased this protein’s phosphorylation (Figure 8A,C).

In SIHA cells, E2, G36, butolame, and pentolame decreased the phosphorylation levels of c-fos compared to the vehicle. Concerning the 17β-AEs, the compound that significantly increased c-fos phosphorylation was prolame. On the other hand, butolame decreased this effect compared to the vehicle and the prolame treatment (Figure 8A,C).

In the combined regimens, E + G treatment showed a similar trend compared to G36 treatment alone. Interestingly, in combination with G36, both prolame and butolame increased c-fos phosphorylation by 31 and 22%, respectively, relative to the vehicle. Notably, the combination of Pent + G markedly reduced the phosphorylation of this protein by 20% with respect to the vehicle (Figure 8A,C).

## 4. Discussion

Exposure to hormonal compounds has long been known to be associated with cervical and breast cancers, to differing extents [49,50,51,52]. Thus, we investigated different synthetic analogs of E2, focusing on their use in HRT without promoting the development and sustained proliferation of cervical and breast cancer cells. In addition, the activation of GPER1 is strongly associated with estrogen signaling and the proliferation of both types of cancer. Our group has studied the effects and mechanism of action of estradiol-like compounds known as 17β-AES in different in vivo models [21,23,24,25]. However, their interactions with GPER1 and their possible proliferative effects in various types of breast cancer cells or cervical cancer cells had not previously been determined. Thus, this study aimed to perform molecular simulation studies of 17β-AEs with GPER1, and corroborate their proliferative effects in cancer cell lines to assess whether these compounds represent potential therapeutic options for HRT in women going through menopause.

In the present study, we performed molecular coupling studies of GPER1 as a preliminary interaction with three 17β-AEs and compared them with already known agonists (E2 and G1) and antagonists (G15 and G36). Once we confirmed this hypothesis, these compounds’ proliferative effects were evaluated in relation to two types of cancer cells that are frequently associated with HRT-related female cancer.

For the in silico analysis that we employed, previous models of binding sites were established in other studies [53,54,55,56]. In addition, we used docking studies, since they have been widely used to identify ligands that may have a therapeutic use [57,58]. In this regard, molecular docking analysis showed that the E2, G36, G15, and G1 compounds and the 17β-AEs bound to GPER1 with different free energy values (Table 2).

Various studies have reported different sites of GPER1 interaction with distinct molecules. In this regard, Lappano et al. (2012) reported that, in the binding pocket of GPER1, the π–π interactions between the PHE:206 and PHE:208 and GPER ligands, such as tamoxifen and fulvestrant, are essential for their binding [56]. Other recent work by Méndez-Luna et al., 2015, also reported that PHE:208 is an important motif for recognizing G1, G15, G36, and E2 by GPER1. Moreover, the authors also described an aromatic cluster composed of PHE:206, PEH:208, PEH:223, and PHE:278, which they also classified as a vital recognition site for different ligands of GPER1 [54]. Notably, it has been reported that the PHE:206 and PHE:208 residues are found in the extracellular helices of GPER1, which have even been associated with the binding of other ligands such as calixpyrrole derivatives [59]. The results of the present work also demonstrated that PHE:206 formed stabilizing π interactions with G1, G15, E2, and prolame. In addition, PHE:208 also displayed hydrophobic interactions with E2 and prolame. Our in silico studies suggest that prolame might join GPER1 in a hydrophobic cavity, similarly to other ligands such as G1, G15, and E2. The results found here are in agreement with previously reported studies [56]. These articles serve to validate our research. The results we obtained from the in silico studies partially explain why prolame has E2-like effects in inducing cell viability proliferation in two cancer cell lines. The high affinity of prolame supports the idea that it presented ΔG values to E2. The docking analysis data revealed that the interactions of prolame and E2 with GPER1 involved almost the same amino acids and sites.

In the first instance, we can consider the fact that prolame probably has more interaction sites with GPER1 than butolame or pentolame. Second, conformational changes induced by prolame and E2 might be similar at the cellular level, and this might lead to the recruitment of coactivating agents in the same way. However, studies confirming these assumptions and identifying the proteins associated with cell proliferation will be the subject of future studies. The cell proliferation and cell cycle results observed in both cell lines support this hypothesis (Figure 4 and Figure 5).

On the other hand, the proposal that GPER1 has different binding sites and acts with different ligands has been suggested by previous studies. As Mendez-Luna et al. stated, it is not completely clear which amino acid residues may be involved in controlling the signal transmission that could explain the biological activity of GPER1 and the pharmacological interaction evoked by antagonists or agonists. Nevertheless, different binding poses and structural conformations of GPER1, favored by its interaction with distinct ligands, may also activate its signaling [54].

In the case of butolame, we did not find shared amino acid residues with those found in the other compounds tested. This indicates that its interaction with GPER1 might occur in another cavity. Interestingly, pentolame showed four amino acid motifs similar to G36: two of them were hydrophobic interactions (threonine (THR) 149 and ARG: 164), one corresponded to a π interaction (PHE: 153), and the other was an alkyl interaction (PHE: 168).

On the other hand, to corroborate the biological activity of the 17β-AEs, we used two types of cell lines (MCF-7 and SIHA) where the presence of GPER1 was previously reported [60,61]. In the cell viability assays, we found that E2 induced significant proliferation in MCF-7 cells, as described in previous reports [62]. Prolame also exhibited a concentration-response behavior that induced the cell viability, similar to E2; this correlates with our hypothesis that prolame is the 17β-AE that possesses E2-like actions, in its ability both to bind to GPER1 and to induce cell proliferation. These effects may be due to the hydrophobic cavity where prolame interacts with the receptor.

In contrast, butolame reduced cell proliferation, and pentolame did not appear to have significant effects on inducing breast cancer cell viability. Remarkably, butolame did not have molecular interactions like those of the agonists and antagonists with which it was compared (G36, G15, G1 and E2). On the other hand, as already mentioned, pentolame shared many of the interactions that G36 exhibits with GPER1. Notably, prolame has three methylene substituents and acts similarly to E2 to induce cell viability in breast cancer cells, as compared to butolame (four methylene groups) or pentolame (five methylene groups). Thus, methyl groups present at carbon 17 on the side chain of the base structure of E2 might promote an agonist pharmacological interaction. Nevertheless, based on the cell viability studies, we can assume that pentolame could act as a weak antagonist.

Regarding the effect of G36 on the viability of MCF-7 cells, we found that this compound partially reduced the viability of this cell line, in agreement with previously published data [63]. Therefore, the addition of G36 to E2 or either of the 17β-AEs independently reversed the cell proliferation induced by the compounds. This biological effect is consistent with the addition of a GPER1 antagonist such as G1, which blocks the effects of E2 in cancer cells [64]. Interestingly, the effect of combining pentolame with G36 at high doses (1 × 10^−9^ M) is similar to that of G36 administered individually. Because pentolame shares binding sites with G36, this finding supports our hypothesis that pentolame has a weak antagonistic character, and is more similar to G36 (Table 1).

We also found that E2 induced an increase in cells in the G2/M phase; this finding is in agreement with the induction of cell proliferation and cell division that has previously been described [62]. Corroborating the viability studies, it can be observed that the exposure of breast cancer cells to G36 decreases the percentages of cells in the G2/M phase. Interestingly, the administration of 17β-AES also diminished the percentage of cells in this phase, but the percentage was significant when they were in the presence of G36. This is an expected effect according to our docking studies, because G36 has a higher affinity for the receptor. Thus, G36 displaces or interferes with the action of another ligand.

To elucidate the possible mechanisms that the 17β-AEs present in the cell cycle, we need to analyze the protein expression of different cyclins or cyclin inhibitors involved in its control. For instance, the literature reports that cyclin D1 is a target regulated by estradiol in MCF-7 cells [65]. Our results showed that this hormone increased the percentage of cells in the G2/M phase; thus, the expression of cyclin D1 would be a good candidate for evaluating a possible mechanism. Therefore, we can assume that prolame could act by similar mechanisms; however, this would have to be corroborated by different protein studies.

Regarding butolame and pentolame, we did not observe significant changes in the different phases of the cell cycle in comparison with the Vh or E2. It is important to note that this is the first exploratory study of the effects of 17β-AEs in concentrations similar to the physiological ranges of E2, so there is no previous information regarding their impact on the modulation of the cell cycle; however, these results will lay the foundations for further research.

On the other hand, the results showed that E2 induced c-fos phosphorylation of the MCF-7 cells, which is in concordance with the study of Maggiolini et al., 2004, who reported that c-fosis is a key protein involved in the proliferation of breast cancer cells by the regulation of AP-1 signaling [66]. Moreover, in the present study, we observed that G36 significantly inhibited the phosphorylation of this protein with respect to E2. Prolame, butolame, and pentolame did not show differences with respect to the effect exhibited by E2.

Concerning the combined schemes, the combination of E2 or prolame with G36 increased c-fos phosphorylation compared with G36 alone. This strengthens the assumption that prolame acts like E2. The addition of G36 to butolame inhibited c-fos phosphorylation induced by exposure to this hormone, but this effect was not observed with the other 17β-AEs. Instead, the Pent + G combination significantly increased c-fos phosphorylation compared to the other treatments.

Different studies have reported that the phosphorylation of c-fos may be supported by the classic activation of the ERα and its consequent activation of the activating protein-1 (AP-1) transcription factor in MCF-7 cells [67]. Even though the combination of Pent + G promoted the phosphorylation of this protein at 6 h, cell viability decreased after 72 h of exposure. Pentolame could be interacting with ERα to induce c-fos phosphorylation because we did not include a specific ERα antagonist. Thus, it will be essential to evaluate the effects of combinations of inhibitors on different pathways, such as MAPK and Hippo/YAP/TAZ, among others, as well as in other types of breast cancer; this will offer a global overview of the effects of 17β-AEs in breast cancer.

In SIHA cells, we found that E2 had a slight effect on the induction of cell proliferation, a finding similar to that reported by Ruutu et al., 2006 [68]. Regarding this last point, we did not find related literature on the effects of G36 on cervical cancer cells. Regarding the combinations of E2 or the 17β-AEs with G36, it is notable that all of them tended to increase cell proliferation with respect to the treatment with independent compounds, but this was not significant. The biggest changes were observed in the combined regimens and generally in the highest concentrations.

G36 induced an increase in the sub-G1 peak, indicating that this compound induced cell death. As G36 is an antagonist of E2, this suggests that, rather than promoting cell proliferation, E2 protects against cell death. E2 tended to increase the percentage of cells in the G2/M phase, as has been reported in another type of cervical cancer cell line [69]. Meanwhile, butolame and prolame showed effects similar to E2 on the G2/M phase, but pentolame had no effect. In the combined schemes, the three 17β-AEs protected against cell death induced by G36 to a greater extent than E2. However, it is noteworthy that pentolame in combination with G36 had a similar effect to G36 alone. This effect is perhaps simply a consequence of the selective GPER1 antagonist G36, which showed high affinity in the silico studies.

Notably, prolame induced the phosphorylation of c-fos at a higher level than butolame, pentolame, and E2. Interestingly, the combination of prolame and butolame with G36 significantly increased the phosphorylation of c-fos, suggesting that this protein activates anti-apoptotic pathways. However, the combination of pentolame with G6 seems to inhibit c-fos phosphorylation, to a greater extent than G36 alone. Thus, G36 inhibits the phosphorylation of c-fos, which triggers the cell death in this line; however, as this is not resolved by pentolame treatment, both phenomena might be independent. Until now, we have not found any studies that discuss the role of GPER1 in the activation of c-fos in cervical cancer cells. However, we are aware that to determine the effects of GPER1 in the activation of different oncogenes in cervical cancer, it would be necessary to carry out complementary studies about the mechanisms of action of the 17β-AEs, both alone and in combination with different inhibitors of GPER1, in the signaling pathways involved in transactivation with GPER1.

In this sense, our results demonstrated more significan changes in cervical cancer cells (SIHA cell line) than in breast cancer cells (MCF-7) in the phosphorylation of c-fos. Based on the assumption that the MAPK pathway can induce c-fos, and on the fact that the overexpression of the epidermal growth factor receptor (EGFR) has been reported in cervical cancer cells [70], we suggest that the modulation of c-fos is not only dependent on GPER1 but also on EGFR. To corroborate this proposal, it is important to conduct studies using combinations with EGFR or MAPK inhibitors, such as gefitinib or PD98059.

## 5. Conclusions

The recognition and functional characterization of novel compounds that act as ligands of GPER1 represents a valuable means of further dissecting this receptor’s pharmacology, and better understanding how its functions are elicited in different types of cancer.

Here, we demonstrated that 17β-AEs might bind to GPER1 based on the prediction of in silico studies carried out in this work. In addition, we reported that 17β-AEs have different effects on breast and cervical cancer cells.

We observed that prolame is the compound that is the most structurally similar to E2, and which might act most similarly to E2. As a final biological event, prolame induced the cell proliferation of both types of cancer cell lines used. Thus, it would be interesting to evaluate the effects of prolame in different pathological contexts, such as autoimmune and inflammatory diseases, or in cardiovascular models where its pharmacological effects could be demonstrated.

In addition, based on our results, we suggest that the 17β-AEs have estrogenic activities mediated by their interaction with GPER1, according to the prediction of the in silico studies carried out in this work. In addition, since neither butolame nor pentolame showed significant differences in inducing cell viability or in modifying breast and cervical cancer cell cycles, both compounds could be promising therapeutic options for use in HRT. Using butolame or pentolame might allow us to avoid the risk of promoting the progression of breast or cervical cancer. However, it is necessary to carry out different complementary in vitro and in vivo experiments that further elucidate their effects on cancer.

## Figures and Tables

**Table 1:** Amino acid residue interactions between G36, G1, G15, E2, and 17β-AEs with GPER1.

	Type of Interaction			
Compound	Van der Waals	Hydrogen Bonds	Pi-Interactions	Alkyl
G36	ARG: 164 THR: 149		ALA: 23 PRO: 24 PHE: 146 TRP: 150 PHE: 153 ILE: 181	PHE: 168 ILE: 229
G1	LEU: 59 GLU: 115 LEU: 119 TM HELIX 2 ASN: 118 ARG: 122 TYR: 123 CYS: 207	GLU: 121	ALA: 204 PHE: 206	TYR: 55
G15	GLN: 54 TYR: 55 GLY: 58 ASN: 118 GLU: 121 ARG: 122 TYR: 123 THR: 201 CYS: 205		LEU: 119 PHE: 206	HIS: 307 TM IV
E2	ASN: 118 GLU: 121 PHE: 208	GLU: 115	LEU: 119 PHE: 206	TYR: 55
Prolame	LEU: 59 GLU: 115 HIS: 120 GLU: 121 ARG: 122 PHE: 208	ASN: 118	TYR: 55 LEU: 119 PHE: 206	
Butolame	ILE: 72 GLY: 76 CIS: 318 PRO: 321 LEU: 322		ALA: 347 PHE: 351	VAL: 75 LEU: 79 LEU: 348
Pentolame	ASN: 25 THR: 149 ARG: 164 LEU: 180 ALA: 184	GLN: 20 PRO: 24	TRP: 150 PHE: 153	ALA: 23 PHE: 146 PHE: 168 ILE: 181 ILE: 229

**Table 2:** Free energy values of the docking simulations for G36, G1, G15, E2, and 17β-AEs.

Compound	ΔG (kcal/mol)
G36	−9.2
G1	−8.9
G15	−8.6
E2	−8.5
Prolame	−8
Pentolame	−7.4
Butolame	−7.3

**Table 3:** Cell-cycle phases of the MCF-7 cell line after exposure to E2, G36, and 17β-AEs, either alone or in a combination scheme.

	Sub G1	G1	S	G2/M
Vh	8.2 ± 0.3	66.7 ± 6.9	20.0 ± 4.8	3.06 ± 0.2
E2	10.0 ± 3.0	53.7 ± 5.0	23.5 ± 5.0	13.9 ± 4.0 ^a^
G36	14.9 ± 1.7 ^a,e^	61.2 ± 2.8	18.6 ± 2.8	7.5 ± 0.6 ^b^
Prolame	7.0 ± 1.0 ^c^	63.6 ± 8.5	21.0 ± 8.5	8.0 ± 2.7
Butolame	10.2 ± 0.9	58.3 ± 4.1	21.0 ± 4.1	8.9 ± 1.4
Pentolame	9.8 ± 1.0	60.4 ± 4.2	21.7 ± 4.2	7.9 ± 1.1
E2 + G	9.0 ± 0.7	67.9 ± 9.5	12.0 ± 9.5	6.7 ± 1.4 ^b^
Pro + G	10.3 ± 2.2	60.0 ± 4.7	20.1 ± 4.7	2.2 ± 1.3 ^b,d^
But + G	7.3 ± 0.1	68.6 ± 14.0	16.6 ± 14.0	7.4 ± 2.6 ^b^
Pent + G	8.0 ± 0.8	66.4 ± 9.7	18.7 ± 9.7	6.6 ± 2.3 ^b^

Statistical differences code: ^a^ vs. Vh; ^b^ vs. E2; ^c^ vs. G36; ^d^ vs. E + G; ^e^ vs. prolame.

**Table 4:** Cell-cycle phases of the SIHA cell line after exposure to E2, G36, and 17β-AEs, either alone or in a combination scheme.

	Sub G1	G1	S	G2/M
Vh	7.1 ± 2.3 ^c^	63.8 ± 8.1 ^c^	19.4 ± 4.1	8.5 ± 9.1
E2	3.04 ± 3.4 ^c^	60.7 ± 2.4 ^c^	21.1 ± 5.7	12.9 ± 6.5
G36	33.6 ± 8.0 ^a,b,d,e,f,g^	43.4 ± 5.6	14.5 ± 2.2	4.9 ± 3.5
Prolame	3.8 ± 2.3 ^c^	63.0 ± 1.2 ^c^	20.9 ± 6.4	10.7 ± 5.9
Butolame	7.8 ± 2.3 ^c^	57.3 ± 1.2	21.7 ± 6.4	11.4 ± 5.9
Pentolame	3.3 ± 0.3 ^c^	64.0 ± 6.2 ^c^	19.1 ± 4.3	8.2 ± 8.9
E2 + G	17.0 ± 1.2 ^b,c^	52.4 ± 10.0	12.8 ± 3.3	6.9 ± 9.2
Pro + G	5.8 ± 5.7 ^c^	59.5 ± 3.1	22.0 ± 5.1	11.0 ± 4.5
But + G	5.6 ± 5.6 ^a,c^	61.0 ± 4.5 ^c^	20.9 ± 4.4	10.8 ± 5.6
Pent + G	5.2 ± 3.5 ^c^	64.8 ± 5.6 ^c^	18.8 ± 3.7	6.6 ± 2.3

Statistical differences code: ^a^ vs. Vh; ^b^ vs. E2; ^c^ vs. G36; ^d^ vs. E + G36; ^e^ vs. prolame; ^f^ vs. Pro + G36; ^g^ vs. Pent+ G36.

## Data Availability

Regarding the availability of data, these can be requested by writing to the corresponding authors.

## Supplementary Materials

The following supporting information can be downloaded at: https://www.mdpi.com/article/10.3390/ijerph191912361/s1, Figure S1: Analysis of the phosphorylation of c-fos at 1 and 6 h in MCF-7 and SIHA cells after the exposure to E2 1 × 10^−9^ M (1 nm). Histograms of (**A**) MCF-7 and (**B**) SIHA cell lines. MFI values for each treatment are shown. Median fluorescence intensity (MFI) of c-fos in (**C**) MCF-7 and (**D**) SIHA cells are displayed.
