# Supplementary material for: The Interplay of GPER1 with 17β-Aminoestrogens in the Regulation of the Proliferation of Cervical and Breast Cancer Cells: A Pharmacological Approach
Source: Int J Environ Res Public Health. 2022 Sep 28;19(19):12361. doi: 10.3390/ijerph191912361 (PMC9566056; doi:10.3390/ijerph191912361)
Supplement: Supplementary file 1 [file ijerph-19-12361-s001.zip › ijerph-1882546-supplementary.pdf]

Supplementary Material

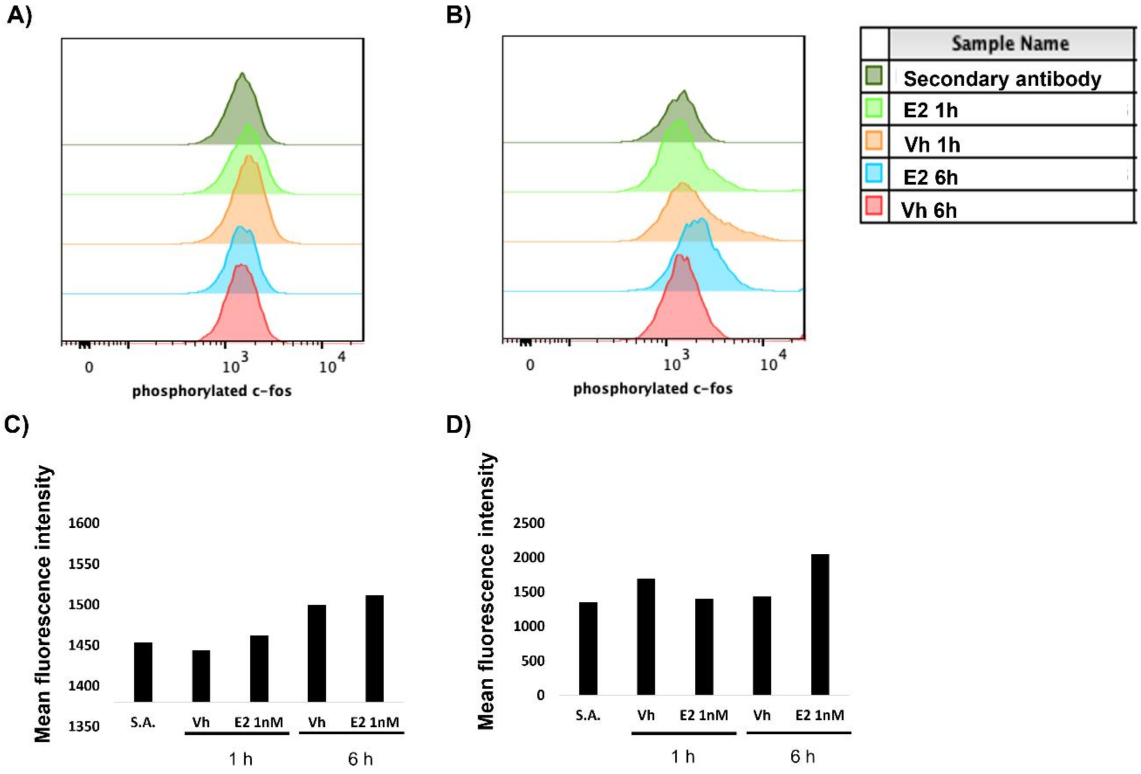

Figure S1. Analysis of the phosphorylation of c-fos at 1 and 6 h in MCF-7 and SIHA cells after the exposure to E2 1 x 10^{-9} M (1nM). Histograms of A) MCF-7 and B) SIHA cell lines. MFI values for each treatment are shown. Median fluorescence intensity (MFI) of c-fos in C) MCF-7 and D) SIHA cells are displayed.
